# Automated interpretation of PD-L1 CPS based on multi-AI models integration strategy in gastric cancer

**DOI:** 10.3389/fimmu.2025.1614099

**Published:** 2025-08-06

**Authors:** Ting Han, Meng Zhuo, Ziyu Song, Peilin Chen, Shiting Chen, Wei Zhang, Yuanyuan Zhou, Hong Li, Dadong Zhang, Xiaolin Lin, Zebing Liu, Xiuying Xiao

**Affiliations:** ^1^ Department of Oncology, Renji Hospital, School of Medicine, Shanghai Jiao Tong University, Shanghai, China; ^2^ Department of Pathology, Renji Hospital, School of Medicine, Shanghai Jiao Tong University, Shanghai, China; ^3^ Product Development Department, SODA Data Technology Inc., Shanghai, China; ^4^ Department of Clinical and Translational Research, 3D Medicines Inc., Shanghai, China; ^5^ School of Pharmacy, East China University of Science and Technology, Shanghai, China; ^6^ State Key Laboratory of Systems Medicine for Cancer, Shanghai Cancer Institute, Renji Hospital, Shanghai Jiaotong University School of Medicine, Shanghai, China

**Keywords:** PD-L1, CPS, gastric cancer, automated scoring, artificial intelligence

## Abstract

**Introduction:**

Programmed cell death ligand-1 (PD-L1) combined positive score (CPS) evaluation plays a pivotal role in predicting immunotherapy efficacy for gastric cancer. However, manual CPS assessment suffers from significant inter-observer variability among pathologists, leading to clinical inconsistencies. To address this limitation, we developed a deep learning-based artificial intelligence (AI) system that automates PD-L1 CPS quantification for patients with gastric cancer (GC) using whole slide images (WSIs).

**Methods:**

We developed a deep learning-based artificial intelligence (AI) system that automates PD-L1 CPS quantification for patients with gastric cancer (GC) using whole slide images (WSIs). Our pipeline firstly employs a dual-network architecture for tumor region detection: MobileNet for patch-level classification and U-Net for pixel-level segmentation. Followed by a YOLO-based cell detection model to compute PD-L1 expression on different cells for CPS calculation. A total of 308 GC WSIs were included, including 210 in the internal cohort and 98 in the external cohort. Within the internal cohort, 100 WSIs were utilized for the model development, while the remaining 110 WSIs served as an internal testing set for comparative analysis between AI-derived CPS values and pathologist-derived reference standards.

**Results:**

The AI-derived CPS demonstrated strong concordance with expert pathologists’ consensus in internal cohort (Cohen’s kappa = 0.782). Furthermore, the AI-based CPS prediction pipeline was evaluated for its performance in the external cohort, and showed robust performance (Cohen’s kappa = 0.737).

**Discussion:**

Our system provides a standardized decision-support tool for immunotherapy stratification in GC management, demonstrating potential to improve CPS assessment reproducibility.

## 1 Introduction

According to 2022 Global Cancer Statistics (1, 2), gastric cancer is the world’s fifth-most prevalent cancer and a major cause of cancer-related death. It is characterized by high heterogeneity and poor prognosis. In recent research, immune checkpoint inhibitors (ICIs), particularly programmed death - ligand 1 (PD-L1) inhibitors, have garnered substantial attention. Immunotherapy, with ICIs at its core, has emerged as a widely adopted approach in treating a spectrum of cancers, including lung cancer, colorectal cancer, liver cancer, and gastric cancer. Multiple prospective, multicenter clinical randomized controlled trials have confirmed that the combination of chemotherapy and ICIs can significantly improve the prognosis of advanced gastric cancer compared with chemotherapy alone. Studies such as Checkmate-649 (3), ORIENT-16 (4), and RATIONALE 305 (5) have established the important role of combined ICI therapy in advanced gastric cancer, which can bring significant survival benefits to some patients.

Despite significant advancements in immunotherapy, its efficacy remains limited to a subset of patients (6). Identifying potential responders who could benefit from this treatment and achieve prolonged survival is therefore of paramount importance in clinical practice. Currently, the CPS system serves as a primary evaluation metric, quantifying the expression level of PD-L1 protein on tumor and immune cell surfaces as a percentage value. Clinically, a higher CPS score likely correlates with increased tumor sensitivity to immunotherapy and predicts better therapeutic outcomes, making it a crucial indicator for clinical decision-making in immunotherapy administration (7, 8). The 2024 CSCO guidelines have made significant updates to the immunotherapy section for gastric cancer, introducing refined stratification based on PD-L1 CPS for the first-line immunotherapy of HER2-positive gastric cancer. However, the current practice of manual CPS calculation for PD-L1 expression assessment presents substantial challenges, primarily due to issues with reproducibility and consistency among pathologists’ evaluations (9).

The integration of artificial intelligence (AI) with digital pathology has catalyzed transformative innovations in diagnostic medicine. Advanced deep learning architectures, including convolutional neural networks (CNNs) and vision transformers, have been engineered to revolutionize pathological workflows by enabling precise tissue segmentation (10), automated metastasis detection (11), and AI-driven prognostic prediction (12). These innovations demonstrate remarkable diagnostic concordance with human pathologists across multiple clinical scenarios (13). Several AI solutions have been developed to assist pathologists in accurately scoring the PD - L1 (Dako 22C3) TPS in non-small cell lung cancer (14, 15), and these solutions demonstrate clinical-grade diagnostic reliability in supporting pathological evaluations. Even when it comes to the more complex interpretation of the (Dako 22C3) CPS, studies suggest that AI models can help reduce discrepancies among pathologists in the context of breast cancer and urothelial carcinoma (16, 17). These AI systems have been shown to enhance both consistency and reproducibility in clinical practice, thereby improving the overall reliability of pathological assessments.

However, the first step in most of these AI-based methods for quantifying tumor markers typically requires segmenting the tumor region using a semantic segmentation model, which is relatively time-consuming. Additionally, for some tumor samples, distinguishing between tumor regions and non-tumor areas (such as normal epithelial tissue, glands, etc.) based solely on immunohistochemistry (IHC) images presents certain challenges. Achieving higher accuracy often requires the integration of multi-dimensional AI algorithms (18).

In this study, to automatically calculate the CPS in gastric cancer, and to improve the efficiency of analysis, we propose an AI-based whole-slide analysis pipeline. The proposed pipeline integrates a pixel-level segmentation model for tumor region delineation with a patch-level classification model for enhanced tumor recognition. Subsequently, a YOLO algorithm was employed to identify target cells for PD-L1 quantification. The primary objective of this study was to develop and evaluate an integrated pipeline to support standardized CPS assessment in gastric cancer diagnostics, with the goal of establishing a framework for automated, AI-assisted clinical CPS evaluation. This framework aims to assist pathologists in CPS calculation and provide a foundation for screening patients who may be suitable for immunotherapy.

## 2 Materials and methods

### 2.1 Materials

A total of 210 formalin-fixed, paraffin-embedded, anonymized samples from patients diagnosed with gastric cancer were collected from 3DMed Clinical Laboratory (accredited by CAP and CLIA) as model development and internal test cohort in this study. Among these, 100 samples were used to develop the deep learning (DL) models, while the remaining 110 samples constituted a held-out internal test set to evaluate the AI-based CPS prediction pipeline performance. Besides, 98 external samples were obtained from Shanghai Renji Hospital and used as external cohort to test the generalization ability of the AI-based pipeline. All the samples were prepared and stained using the PD-L1 IHC 22C3 pharmDx assay (Dako, Carpenteria, CA, USA) on the Dako Autostainer Link 48 platform, according to the manufacturer’s protocol. After the completion of section staining, all the tissues on the stained sections were scanned and digitized at 20× magnification (0.475 μm/pixel) as WSIs using a KFBIO FK-Pro-120 slide scanner. The exclusion criteria for samples include severe tissue folds/tears, strong nonspecific staining, the presence of large bubble issues, among others. The interpretations of CPS values were performed by two trained pathologists (PD-L1 22C3 assay certified) under double-blinded conditions. To ensure the precision and reliability of the DL model, only WSI samples with concordant diagnoses from two pathologists were retained as ground truth for subsequent comparison with DL model outputs.

### 2.2 Overall workflow of CPS prediction

**Figure 1:**
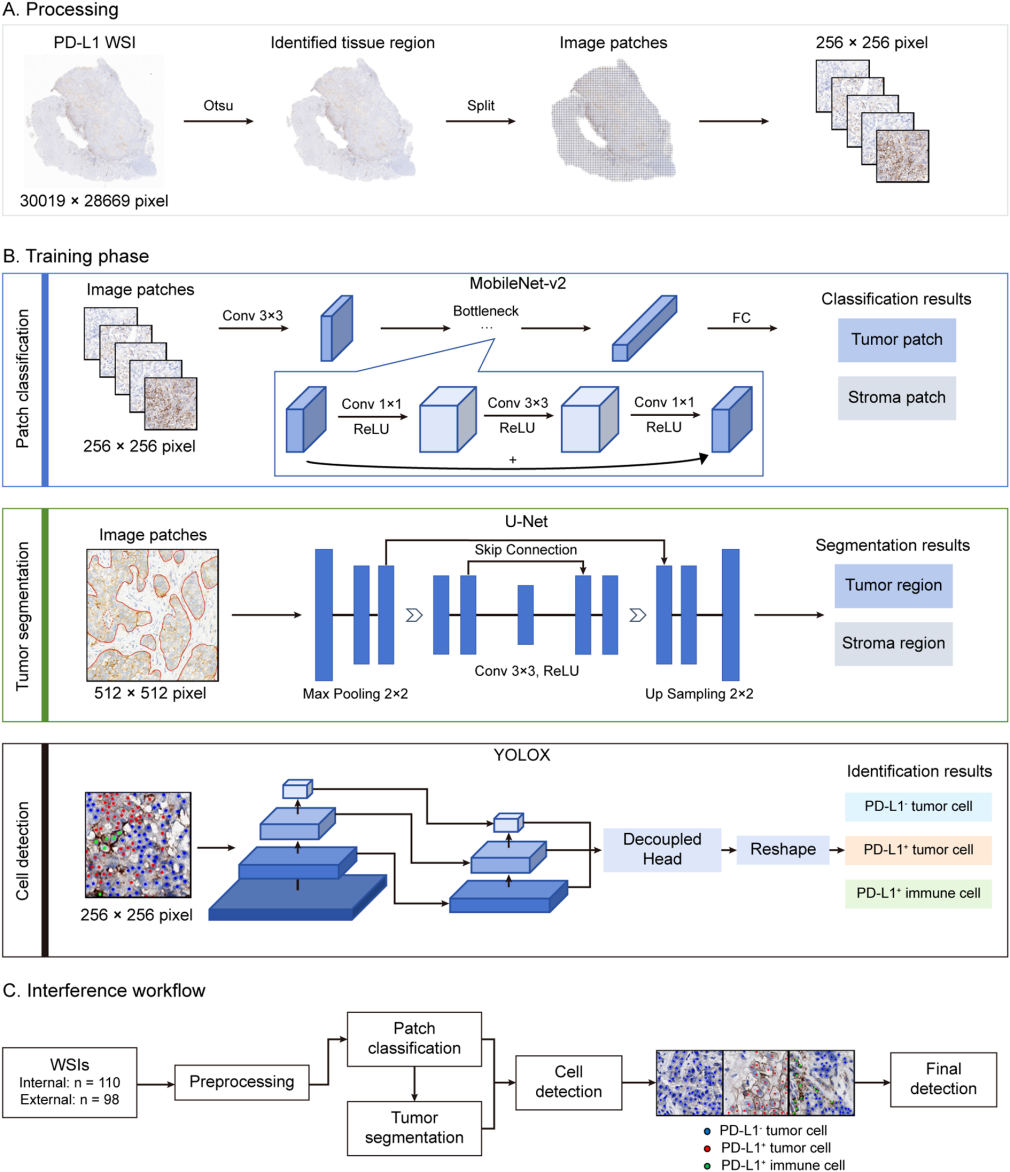
AI pipeline for CPS evaluation. Overview of the proposed AI pipeline in this study. **(A)** Preprocessing: prior to DL models prediction, tissue regions within WSIs were automatically localized through Otsu’s thresholding, followed by dividing into non-overlapping 256×256 pixel patches at 20× magnification. **(B)** Model training. The patch classification model (MobileNet v2), tumor segmentation model (U-Net) and cell detection model (YOLOX) were trained on the corresponding annotation datasets. **(C)** The fully automated pipeline for CPS prediction. After preprocessing, the patches were input into the patch classification model to identify tumor-containing patches. These tumor-containing patches were also fed into the tumor segmentation model to obtain the segmented tumor regions. By combining the results from the above two models, the output patches were input into the cell detection model. The resulting cells were used for CPS calculation.

To achieve automated prediction of PD-L1 expression in gastric cancer, we constructed an AI algorithm-based prediction pipeline. This pipeline integrates sequential deep learning models operating without manual intervention during testing. Each model was individually trained and validated with corresponding annotated data. All data annotations were performed by pathologists using an in-house developed software (APTime, developed by 3D Medicines Inc.). The fully automated pipeline for CPS prediction, as illustrated in **Figure 1C**, initiates with tissue localization. It was performed on WSIs using Otsu thresholding on grayscale-converted slides at 0.625 × magnification (**Figure 1A**). Otsu preprocessing significantly enhanced computational efficiency by eliminating redundant patch classification across non-informative background regions. During the subsequent model prediction phase, all processing was conducted at a magnification of 20 × (0.475 μm/pixel). Identified tissue regions were then partitioned into non-overlapping patches with 256 × 256 pixel size. A trained MobileNet-v2 patch classifier then categorized these patches as either tumor-containing or non-tumor-containing. To refine tumor regions identification, patches classified as tumor by the MobileNet-v2 were then processed by a trained U-Net model for pixel-level segmentation of tumor versus non-tumor regions. Only patches exhibiting consensus tumor regions (those classified as tumor by MobileNet-v2 and simultaneously segmented as tumor by U-Net) were retained for subsequent tumor cell analysis. At last, the trained YOLO-based detector performed triple-task recognition: detection of (1) PD-L1^+^ tumor cells, (2) PD-L1^−^ tumor cells in the tumor regions, and detection of (3) PD-L1^+^ immune cells in tumor-containing patches associated non-tumor regions. The final CPS was calculated based on the cellular counts derived from YOLO detection outputs.

### 2.3 The development of patch classification model

To prepare the training dataset of the patch classification model, the 100 WSIs used for model development cohort were divided into 256 × 256 pixel patches. For samples with extensive tumor regions, pathologists selected those representative tumor patches. For samples containing limited tumor regions, we retained as many tumor-containing patches as possible to incorporate into the training data set. These patches were labeled as either tumor-containing patches or non-tumor patches based on the presence or absence of tumor cells. To maintain class balance and enhance model performance during training, we selected a comparable number of non-tumor patches to tumor-containing patches from each sample, resulting in a final dataset comprising 118,715 tumor-containing patches and 119,476 non-tumor patches (including necrotic areas, normal epithelial regions, stromal regions, etc.).

The dataset was partitioned into training, validation, and test subsets at a 6:2:2 ratio, and to enhance model robustness, we use random flip, rotation, and blur to augment the data during training. We employed the MobileNet-v2 architecture, a lightweight convolutional neural network (CNN) optimized for computational efficiency, as the backbone for patch classification (19). The model leveraged transfer learning through ImageNet pre-trained weights, with strategic fine-tuning: only the final seven layers were unfrozen to adapt domain-specific histopathological features while preserving generic pattern recognition capabilities from pre-training. Following the convolutional layers, the architecture includes a flatten layer and a dense layer (**Figure 1B**).

### 2.4 The development of tumor segmentation model

To prepare the training dataset of the tumor segmentation model, representative patches with 1024 × 1024 pixels size from the 100 WSIs in the model development dataset were selected for pixel-level annotation. We constructed a dataset comprising 3,923 image patches. Among these, 1,929 tumor-containing patches were annotated with pixel-level tumor region labels by pathologists, while 1,994 additional patches containing normal tissues or adjacent non-tumorous tissues were incorporated into the training set, serving as background to enhance the model’s ability to distinguish tumor boundaries.

The datasets were randomly split into training, validation and test set in a ratio of 7:2:1. Data augmentation including random flipping and rotation, and hue, saturation and value change were used during training to avoid overfitting and improve accuracy and generalization ability of the model. Tumor segmentation model was built based on a U-Net structure, with Xception-style block, which consists of separable convolution layer to reduce the number of parameters and accelerate inference (20). The model is a symmetric encoder-decoder architecture with skip connection. In the training procedure, labeled patches were further cropped to 512 × 512 size during training. The DL model was trained to simultaneously segment the tumor area and classify the input region as auxiliary loss. Only the output of the tumor area segmentation task was used to predict the tumor region (**Figure 1B**).

### 2.5 The development of cell detection model

To prepare the training dataset of the cell detection model, representative patches (256 × 256 pixel size) containing PD-L1^+^ and PD-L1^−^ tumor cells, or PD-L1^+^ immune cells, were selected from the 100 cropped WSIs used for model development. We finally constructed a dataset comprising 4604 image patches. On these patches, cells were annotated by experienced pathologists using spots with cell tags and were grouped into PD-L1^+^ tumor cells (85,659), PD-L1^+^ immune cells (19,434), and PD-L1^−^ tumors cells (130,512). When annotating PD-L1-positive cells, we labeled cells exhibiting diverse expression intensity levels (including strong, moderate, and weak). For immune cells, we also labeled various morphological forms of both lymphocytes and macrophages.

We built the cell detection model based on the YOLOX (21), which can directly classify, locate, and count the objects on the input patches (**Figure 1B**). In the data augmentation step, the same strategies as U-Net and mosaic and mixup were applied.

### 2.6 CPS algorithm

CPS is generally calculated by dividing the number of PD-L1 stained cells (including tumor cells, lymphocytes and macrophages) by the total number of viable tumor cells, multiplied by 100. The total formula is shown below:


$$\text{CPS}=\;\frac{\begin{array}{c} Number\;of\;PD-L1\;stained\;cells\\ \left(tumor\;cells,\;lymphocytes,\;and\;macrophages\right) \end{array}}{Total\;number\;of\;viable\;tumor\;cells}\times 100$$

To ensure precise calculation of the CPS, the following criteria must be rigorously applied: 1) PD-L1-positive tumor cells are defined as tumor cells exhibiting partial or complete linear membrane staining within tumor nests, excluding cells in necrotic areas. 2) PD-L1-positive immune cells should be quantified only if they are located within tumor nests or adjacent supporting stroma and maintain direct spatial proximity to tumor cells (within a 0.5 mm radius).

As cells were grouped into PD-L1^+^ tumor cells, PD-L1^+^ immune cells, and PD-L1^−^ tumors cells by cell detection model, the final formula is shown below:


$$\text{CPS}=\;\frac{PD-L1^{+}\text{ tumor cells}+PD-L1^{+}\text{ immune cells}}{PD-L1^{+}\text{ tumor cells}+\;PD-L1^{-}\text{ tumors cells}}\times 100$$

In clinical diagnosis, pathologists approximately distinguish the tumor cell region from other regions firstly at the lower magnification scale and then zoom into the higher magnification for accurate cell counting, and ultimately render a definitive CPS positive/negative assessment. In this study, the final CPS was calculated using counts of PD-L1^+^ tumor cells, PD-L1^+^ immune cells, and PD-L1^−^ tumor cells detected by YOLOX.

The CPS threshold for PD-L1 positivity is defined as ≥ 1. Based on this cutoff, samples were stratified into two distinct subgroups: PD-L1^−^ samples: CPS < 1; PD-L1^+^ samples: CPS ≥ 1.

### 2.7 Evaluation metrics and statistical analyses

This study employed a comprehensive set of evaluation metrics to assess AI model performance, including: Classification metrics (accuracy, precision, recall, specificity, and F1 score); Segmentation metrics (dice coefficient and pixel accuracy); Target detection metrics (Intersection over Union (IoU), Average Precision (AP)). Consistency between AI-calculated CPS values and pathologist assessments was analyzed using confusion matrices and Cohen’s kappa coefficient. The kappa statistic (range: 0 - 1) was interpreted using established clinical benchmarks: slight agreement (0 - 0.2); fair agreement (0.2 - 0.4); moderate agreement (0.4 - 0.6); substantial agreement (0.6 - 0.8); near-perfect agreement (0.8 - 1.0).

All statistical analyses and graphical visualizations were conducted using Microsoft Excel and Python (version 3.9.12), implemented through the PyCharm 2021.3.3 integrated development environment.

## 3 Results

### 3.1 Clinicopathological characteristics of patients

For the 210 specimens utilized in model development and internal validation. As detailed in **Table 1**, both cohorts demonstrated comparable clinicopathological characteristics. The majority of the samples were surgical resection 79% (166/210), and a minority were needle biopsy and others 21% (44/210). All tumor samples were exclusively collected from the stomach. No statistically significant differences were observed between the two groups (all p-values > 0.05).

**Table 1:** Clinicopathological characteristics of gastric cancer samples.

Characteristics	Internal data		*X* ^2^	*P*-value
	Training set (N =100)	Test set (N =110)		
Gender				
Male	57	76	3.298	0.069
Female	43	34		
Age (years)				
≤ 65	64	61	1.588	0.208
> 65	36	49		
Sampling methods				
Surgical Operation	79	87	1.411	0.494
Needle Biopsy	8	5		
Others	13	18		

### 3.2 Performance of patch classification model

**Figure 2:**
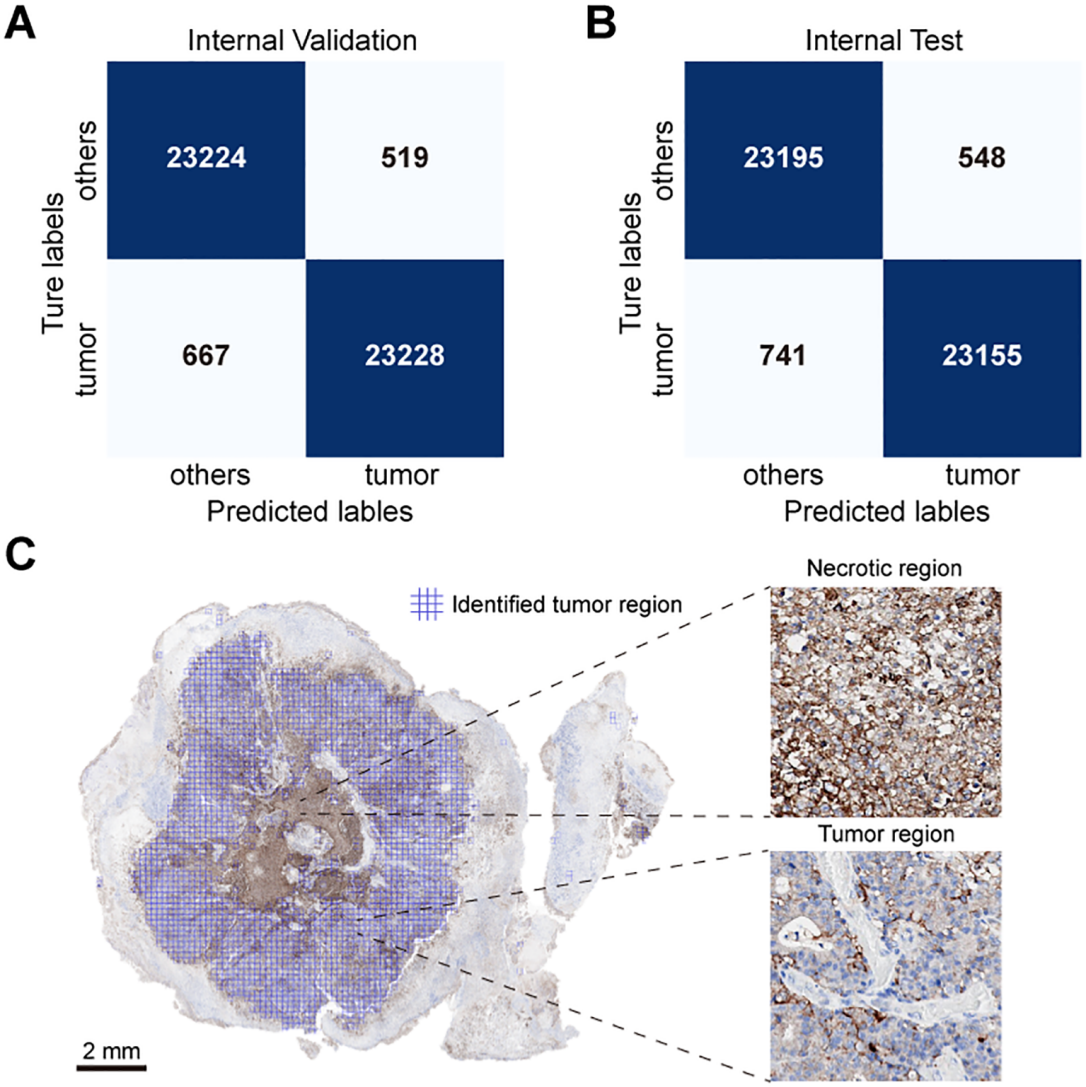
Performance evaluation of classification model. Patch classification results of the MobileNet v2 based on confusion matrix in **(A)** validation set and **(B)** test set. **(C)** Example diagram of the patch classification model results, with two zoomed-in sections demonstrating the model’s ability to exclude necrotic regions and recognize Tumor regions. The blue-tinted regions denote algorithm-identified tumor areas.

To evaluate the PD-L1 expression in the tumor region of a sample, it is essential to accurately localize the tumor area. Considering that patch-level classification models are more efficient in analysis compared to pixel-level segmentation models, and the annotations required for training patch classification models are relatively easier to obtain, we first trained a patch classification model to localize the tumor region. We divided the annotated patches into a training set, a validation set, and an independent test set. The trained model demonstrated high performance on both the validation (**Figure 2A**) and test sets (**Figure 2B**), with accuracy, specificity, and sensitivity all exceeding 97% (**Table 2**). The trained classification model also effectively distinguished stained necrotic regions from tumor regions (**Figure 2C**), thereby eliminating the impact of necrotic regions on PD-L1 evaluation.

**Table 2:** Performance evaluation of classification model.

Statistics	Validation set	Test set
Accuracy	0.975 [0.974, 0.977]	0.973 [0.972, 0.974]
Precision	0.978 [0.977, 0.980]	0.977 [0.976, 0.978]
Recall	0.972 [0.971, 0.974]	0.970 [0.967, 0.971]
Specificity	0.978 [0.977, 0.980]	0.977 [0.976, 0.978]
F1 score	0.975 [0.974, 0.977]	0.973 [0.972, 0.974]

Data was presented as score [95% confidence interval (CI)].

### 3.3 Performance of tumor segmentation model

The tumor patches identified by the patch classification model not only encompass tumor regions but also contain partial stroma areas. Therefore, a segmentation model is further required to distinguish between tumor and stroma.

**Figure 3:**
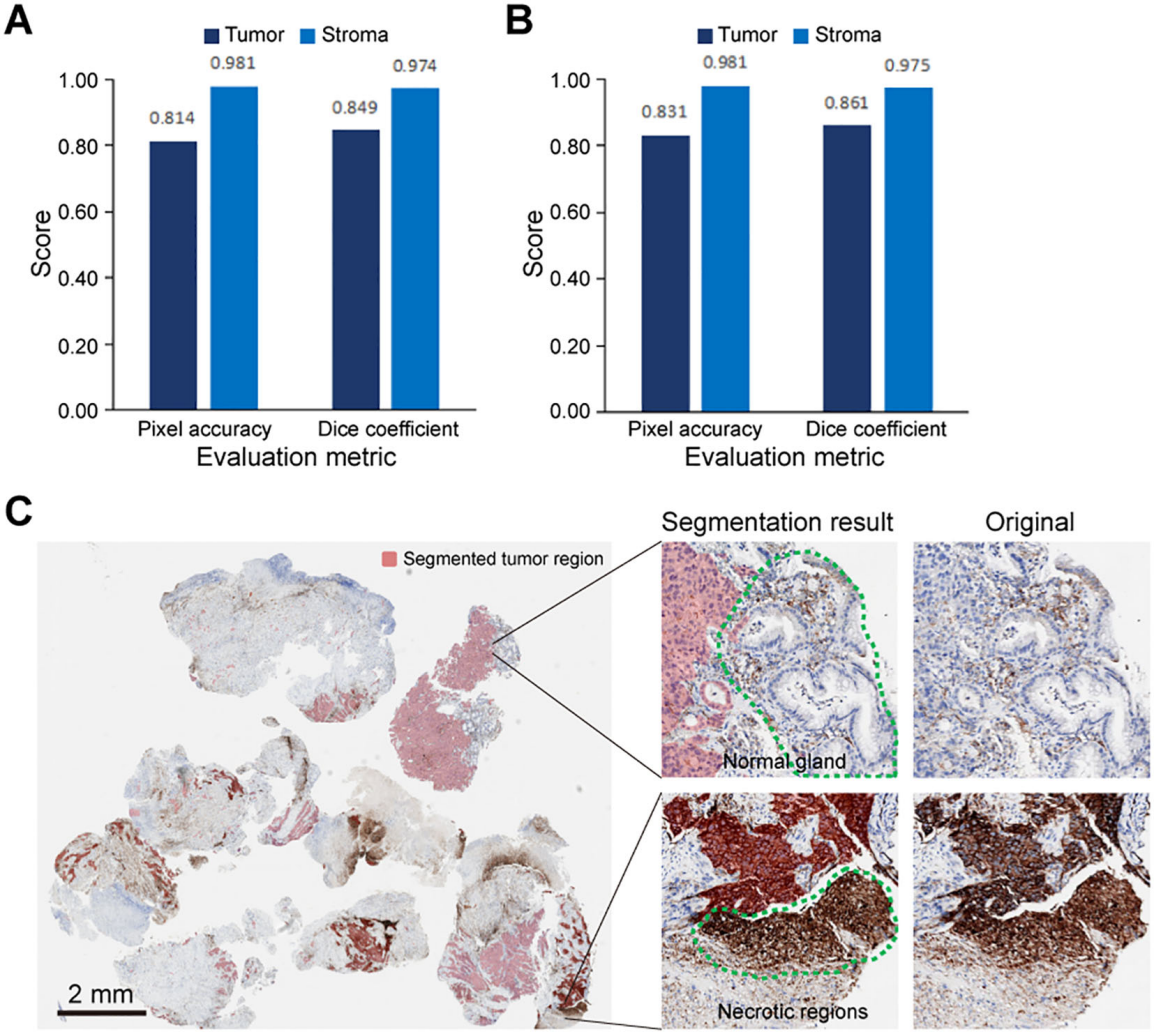
Performance evaluation of tumor segmentation model. The analysis of pixel accuracy and dice coefficient in **(A)** validation set and **(B)** test set. **(C)** An example diagram of the segmentation model result, with the comparison between the segmentation results and the corresponding original patch demonstrating the model’s ability to exclude necrotic regions and normal glandular structures. Green curves delineate manually annotated normal glands and necrotic regions; red contours indicate AI-predicted tumor regions.

In this study, we trained a segmentation model that can distinguish tumor regions from non-tumor regions, which include necrosis and normal epithelium. The model’s performance was evaluated using dice coefficient and pixel accuracy metrics. On both the validation and test sets, the model demonstrated high segmentation performance. Notably, it effectively identified non-tumor regions, with all metrics exceeding 97% (**Figures 3A, B**). Additionally, the segmentation model can accurately distinguished tumor regions from necrotic areas and normal glandular structures (**Figure 3C**).

### 3.4 Performance of model on cell detection

Within our PD-L1 expression evaluation pipeline, precise tumor region localization is followed by quantification of PD-L1^+^ tumor cells, PD-L1^−^ tumor cells, and PD-L1^+^ immune cells within tumor-associated regions. To accomplish this, we developed a deep learning-based cell detection model utilizing the YOLO framework for identification of these three cellular phenotypes. Since immune cells can infiltrate into the tumor region, the calculation of the CPS requires integration with the output of tumor segmentation model. This allows us to distinguish PD-L1^+^ tumor cells, PD-L1^−^ tumor cells, PD-L1^+^ immune cells within the tumor regions, and PD-L1^+^ immune cells within the non-tumor regions for final CPS calculation (**Supplementary Figure S1**).

We evaluated the model’s performance using IoU and AP, with an IoU threshold set at 0.5. The trained cell detection model demonstrated strong performance on both the validation and test sets, achieving AP scores close to 0.900 for true positives (0.889 on the validation set and 0.888 on the test set) (**Table 3**).

**Table 3:** Performance of model on cell detection.

Cell type	AP	
	Validation	Test
TN	0.857	0.866
TP	0.899	0.888
IP	0.869	0.864

TN, tumor negative cells; TP, tumor positive cells; IP, immune positive cells; AP, average precision.

### 3.5 Comparison of consistency between AI pipeline and pathologists

**Figure 4:**
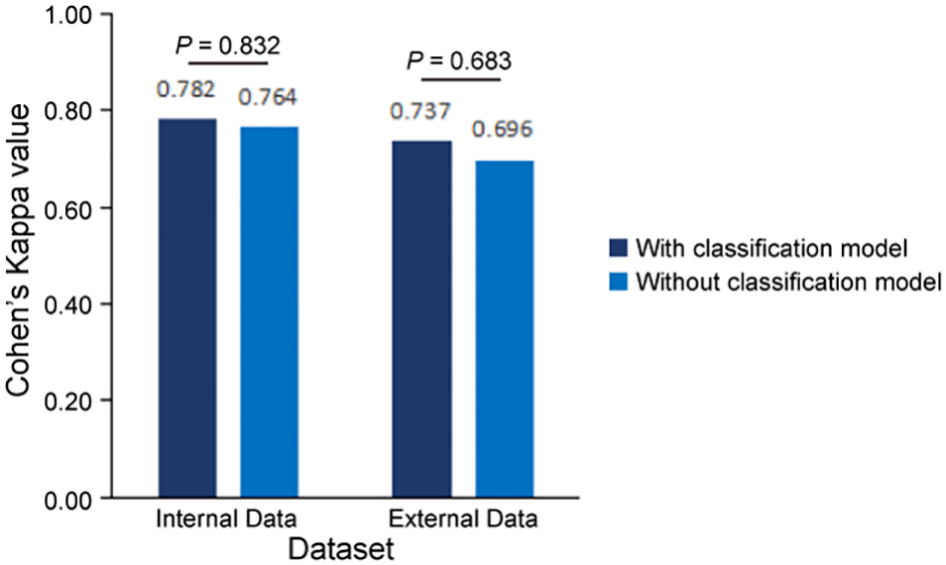
Consistency evaluation between AI pipeline and pathologists. Measures of concordance of combined positive score (CPS) interpretation results between AI pipelines (with and without classification model) and pathologists using Kappa values, all p<0.001. Statistical comparison revealed no significant difference in predictive performance between models with and without the classification module (Internal cohort: p=0.832; External cohort: p=0.683, two-sided Mann-Whitney U test).

To validate the accuracy of our AI-based pipeline, we assessed the agreement between CPS-AI (AI-derived CPS) and CPS-Doc (pathologist-evaluated CPS) using Cohen’s kappa coefficient in internal and external cohorts. First, we evaluated our pipeline on a held-out internal test cohort (n = 110), which was excluded from model training. To further test generalizability, an independent external cohort (n = 98) was introduced. As shown in **Figure 4**, the internal dataset achieved a kappa value of 0.782, demonstrating substantial agreement between model predictions and pathologist-evaluated scores. While the external dataset exhibited a slightly lower value of 0.737, the kappa value remained clinically meaningful, confirming the model’s robustness across diverse datasets.

**Figure 5:**
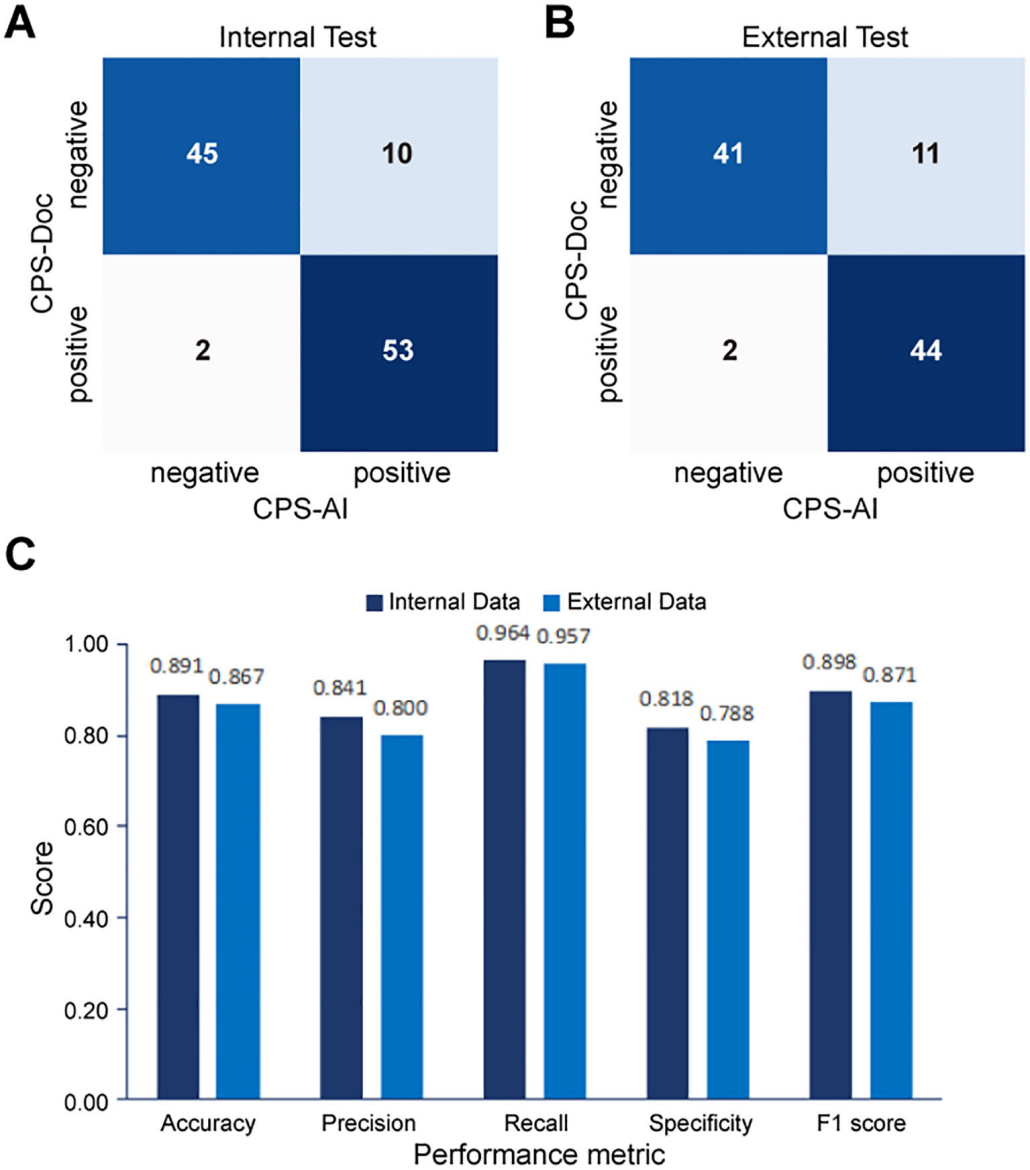
Performance evaluation of the AI pipeline for combined positive score (CPS) prediction. Comparison between CPS predicted by the AI pipeline (CPS-AI) and by doctors (CPS-Doc) in **(A)** the internal validation cohort and **(B)** the external test cohort. **(C)** Histograms of AI models performance in the internal cohort and the external cohort.

To further quantify performance, confusion matrices were generated to compare CPS-AI against CPS-Doc. Using CPS-Doc as the reference standard, we evaluated the CPS prediction accuracy of the AI-based pipeline across multiple metrics. In the internal test cohort, AI-based pipeline achieved an accuracy of 0.882 [95% CI = 0.822 - 0.942], sensitivity of 0.964 [95% CI = 0.929 - 0.999], and specificity of 0.800 [95% CI = 0.725 - 0.875], highlighting its strong discriminative capability (**Figures 5A, C**). Performance remained robust in the external cohort, with retained accuracy and high sensitivity (**Figures 5B, C**). These results collectively underscore the reliability and generalizability of the AI pipeline.

### 3.6 Evaluating combined effectiveness of classification model and segmentation model

In this study, we combined a patch classification model with a region segmentation model to localize tumor areas, where only tumor regions simultaneously identified by both models would proceed to subsequent cell detection. This design offers dual advantages: On one hand, integrating results from both models may enhance the accuracy of tumor region identification. On the other hand, leveraging the higher efficiency of the patch classification model to first roughly localize tumor regions, followed by region segmentation on these pre-selected patches, significantly improves the overall analysis efficiency. Our findings demonstrate that the integrated pipeline incorporating the patch classification model achieved slightly improved consistency with the pathologists compared to the pipeline using only region segmentation with cell detection (**Figure 4**, **Supplementary Figure S2**). Regarding efficiency, the integrated workflow showed 25 - 30% improvement in average processing time per sample (**Table 4**). Particularly for samples with small tumor areas relative to the whole tissue section, a more than twofold enhancement in processing efficiency was achieved (**Supplementary Figure S3**).

**Table 4:** Compare the efficiency of different pipelines in image processing.

Process	Metric	Pipeline with classification	Pipeline without classification
Internal Cohort	Avg Time (s)	136.473	183.761
	Avg Time per Tumor Patch (s)	0.119	0.174
	Avg Time per Unit Area (s/cm^2^)	166.386	210.471
External Cohort	Average Time (s)	176.762	263.655
	Avg Time per Tumor Patch (s)	0.182	0.059
	Avg Time per Unit Area (s/cm^2^)	67.722	101.991

Avg, average.

## 4 Discussion

Immunotherapy with ICIs has now become a new and important treatment option for gastric cancer. Accurate assessment of PD-L1 by pathologists provides essential guidance for selecting gastric cancer patients suitable for ICI therapy. However, current evaluations of PD-L1 by pathologists still lack satisfactory consistency and reproducibility (22). Particularly for assessing the CPS, challenges arise not only from evaluating PD-L1 expression on tumor cells but also on immune cells. Given the vast morphological differences between immune cells and tumor cells, objective CPS quantification poses significant difficulties (23).

With the rapid advancement of image analysis technology, AI-based digital pathology tools are playing an increasingly critical role in pathological diagnosis (24). In clinical practice, pathologists are skilled at qualitative tasks such as localizing tumor regions or excluding nonspecific staining regions, but they are less precise in quantitative counting compared to computational methods. Recent studies have applied deep learning algorithms to develop AI models for assisting various quantitative biomarker assessments, including HER2, Ki67 in breast cancer (25–27), and PD-L1 in lung cancer (14, 28), aiming to improve accuracy and reproducibility. Currently, most PD-L1 expression evaluation AI models focus on evaluating tumor cell expression in lung cancer, with limited research on CPS scoring. However, PD-L1 assessment is clinically relevant across various cancer types and requires consideration of immune cell PD-L1 expression. The AI-assisted CPS diagnostic model for gastric cancer developed in this study effectively addresses this gap.

Most previous AI workflows for biomarker quantification typically involve a segmentation model to identify tumor regions, followed by a nuclues segmentation model, and another model for cell types classification (15–17). These workflows could be time-consuming. Moreover, distinguishing tumor regions solely based on IHC images can be challenging in some cases, leading to suboptimal segmentation accuracy (29). To mitigate this, some studies align corresponding hematoxylin and eosin (H&E) stained WSIs with IHC images, leveraging the richer structural information in H&E images to map tumor regions onto IHC images (30). In this study, our AI model innovatively combines a patch classification model and a tumor segmentation model to localize tumor regions, enhancing both performance and processing efficiency. Only regions simultaneously identified by both models are included in subsequent calculations - akin to pathologists prioritizing consensus regions for biomarker assessment in clinical practice.

The integration of patch classification and region segmentation also improves computational efficiency, particularly for samples with small tumor-to-tissue ratios. Since segmentation models process images at the pixel level and are computationally intensive, restricting segmentation to tumor-containing patches (pre-identified by the classification model) significantly reduces processing time. Moreover, if the classification model achieves high accuracy, the segmentation model can focus solely on distinguishing tumor and stromal regions within these patches, bypassing morphologically ambiguous structures like benign lesions or normal epithelium. While this dual-model approach requires training two tumor localization models, patch-level annotations are relatively easier to obtain, potentially reducing overall labeling efforts.

The study still has some limitations: 1) The study included data from only two institutions, with limited sample size (100 samples for model development) and uniform scanner use. This may lead to decreased model performance when deployed across different hospitals. Future efforts should diversify sample sizes and data sources to enhance the model’s generalizability. 2) Given potential staining variations across different PD-L1 antibody assays, which may reduce model performance or compromise generalizability, application to other PD-L1 antibodies requires model retraining with expanded samples. 3) Although the AI-based CPS prediction pipeline developed in this study is fully automated and demonstrates close concordance with pathologist assessments, inconsistencies persist, highlighting the need for further optimization. These discrepancies are mainly attributable to two factors: Firstly, weakly stained tumor cells were confirmed as a primary source of inconsistency, as faint or incomplete membranous PD-L1 staining occasionally led to their missed detection by the AI system, whereas pathologists successfully identified them through careful microscopic evaluation. Secondly, background interference caused by necrotic debris, mucin deposits, or staining artifacts occasionally generated false-positive signals in the cell detection algorithm, particularly in challenging histological subtypes. Consequently, this pipeline should currently serve as an adjunct tool to assist pathologists in interpretation. 4) The dual-model for tumor localization strategy has room for refinement. Future studies could train models on distinct datasets to simulate pathologists with varying experience, improving localization accuracy.

In summary, the AI pipeline developed in this study demonstrated high consistency with pathologists in internal and external test cohorts, along with efficient image processing. By enabling precise cell quantification and tumor region delineation on WSIs, these AI pipelines enhance model interpretability and assist pathologists in reviewing and verifying results, minimizing oversights or misjudgments, meanwhile enhancing pathologists’ trust in the AI model. This approach holds significant potential for clinical adoption in PD-L1 CPS assessment for gastric cancer and beyond.

## Data Availability

The original contributions presented in the study are included in the article/**Supplementary Material**. Further inquiries can be directed to the corresponding authors.

## References

[1] Bray F Laversanne M Sung H Ferlay J Siegel RL Soerjomataram I. Global cancer statistics 2022: GLOBOCAN estimates of incidence and mortality worldwide for 36 cancers in 185 countries. CA Cancer J Clin. (2024) 74:229–63. doi: 10.3322/caac.21834, PMID: 38572751

[2] Wang S Zheng R Li J Zeng H Li L Chen R. Global, regional, and national lifetime risks of developing and dying from gastrointestinal cancers in 185 countries: a population-based systematic analysis of GLOBOCAN. Lancet Gastroenterol Hepatol. (2024) 9(3):229–37. doi: 10.1016/S2468-1253(23)00366-7, PMID: 38185129 PMC10849975

[3] Janjigian YY Shitara K Moehler MH Garrido M Gallardo C Shen L. Nivolumab (NIVO) plus chemotherapy (chemo) vs chemo as first-line (1L) treatment for advanced gastric cancer/gastroesophageal junction cancer/esophageal adenocarcinoma (GC/GEJC/EAC): 3-year follow-up from Check-Mate 649. Ann Oncol. (2023) 34:S1254–335.

[4] Xu J Jiang H Pan Y Gu K Cang S Han L. Sintilimab plus chemotherapy for unresectable gastric or gastroesophageal junction cancer: the ORIENT-16 randomized clinical trial[J. JAMA. (2023) 330:2064–74. doi: 10.1001/jama.2023.19918, PMID: 38051328 PMC10698618

[5] Moehler M Kato K Arkenau T Oh DY Tabernero J Cruz-Correa M. Rationale 305: Phase 3 study of tislelizumab+chemotherapy vs placebo+chemotherapy as first-line treatment of advanced gastric or gastroesophageal junction adenocarcinoma. J Clin Oncol. (2023) 41:286. doi: 10.1200/JCO.2023.41.4_suppl.286 40528576

[6] Bintintan V Burz C Pintea I Muntean A Deleanu D Lupan I. Predictive factors of immunotherapy in gastric cancer: A 2024 update. Diagnostics (Basel). (2024) 14:1247. doi: 10.3390/diagnostics14121247, PMID: 38928662 PMC11202567

[7] Xie T Zhang Z Zhang X Qi C Shen L Peng Z. Appropriate PD-L1 cutoff value for gastric cancer immunotherapy: A systematic review and meta-analysis. Front Oncol. (2021) 11:646355. doi: 10.3389/fonc.2021.646355, PMID: 34540656 PMC8440909

[8] Noori M Fayyaz F Zali MR Bashash D. Predictive value of PD-L1 expression in response to immune checkpoint inhibitors for gastric cancer treatment: a systematic review and meta-analysis. Expert Rev Anticancer Ther. (2023) 23:1029–39. doi: 10.1080/14737140.2023.2238896, PMID: 37466449

[9] Robert ME Rüschoff J Jasani B Graham RP Badve SS Rodriguez-Justo M. High interobserver variability among pathologists using combined positive score to evaluate PD-L1 expression in gastric, gastroesophageal junction, and esophageal adenocarcinoma. Mod Pathol. (2023) 36:100154. doi: 10.1016/j.modpat.2023.100154, PMID: 36925069

[10] Ma J He Y Li F Han L You C Wang B. Segment anything in medical images. Nat Commun. (2024) 15:654. doi: 10.1038/s41467-024-44824-z, PMID: 38253604 PMC10803759

[11] Ehteshami Bejnordi B Veta M Johannes van Diest P van Ginneken B Karssemeijer N Litjens G. Diagnostic assessment of deep learning algorithms for detection of lymph node metastases in women with breast cancer. JAMA. (2017) 318:2199–210. doi: 10.1001/jama.2017.14585, PMID: 29234806 PMC5820737

[12] Volinsky-Fremond S Horeweg N Andani S Barkey Wolf J Lafarge MW de Kroon CD. Prediction of recurrence risk in endometrial cancer with multimodal deep learning. Nat Med. (2024) 30:1962–73. doi: 10.1038/s41591-024-02993-w, PMID: 38789645 PMC11271412

[13] Echle A Rindtorff NT Brinker TJ Luedde T Pearson AT Kather JN. Deep learning in cancer pathology: a new generation of clinical biomarkers. Br J Cancer. (2021) 124:686–96. doi: 10.1038/s41416-020-01122-x, PMID: 33204028 PMC7884739

[14] Cheng G Zhang F Xing Y Hu X Zhang H Chen S. Artificial intelligence-assisted score analysis for predicting the expression of the immunotherapy biomarker PD-L1 in lung cancer. Front Immunol. (2022) 13:893198. doi: 10.3389/fimmu.2022.893198, PMID: 35844508 PMC9286729

[15] Wu J Liu C Liu X Sun W Li L Gao N. Artificial intelligence-assisted system for precision diagnosis of PD-L1 expression in non-small cell lung cancer. Mod Pathol. (2022) 35:403–11. doi: 10.1038/s41379-021-00904-9, PMID: 34518630

[16] Lee KS Choi E Cho SI Park S Ryu J Puche AV. An artificial intelligence-powered PD-L1 combined positive score (CPS) analyser in urothelial carcinoma alleviating interobserver and intersite variability. Histopathology. (2024) 85:81–91. doi: 0.1111/his.15176, PMID: 38477366 10.1111/his.15176

[17] Li J Dong P Wang X Zhang J Zhao M Shen H. Artificial intelligence enhances whole-slide interpretation of PD-L1 CPS in triple-negative breast cancer: A multi-institutional ring study. Histopathology. (2024) 85:451–67. doi: 10.1111/his.15205, PMID: 38747491

[18] Chen H Li C Wang G Li X Mamunur Rahaman M Sun H. GasHis-Transformer: A multi-scale visual transformer approach for gastric histopathological image detection. Pattern Recognit. (2021) 130:108827. doi: 10.1016/j.patcog.2022.108827

[19] Sandler M Howard A Zhu M Zhmoginov A Chen L-C. “MobileNetV2: Inverted residuals and linear bottlenecks,” 2018 IEEE/CVF Conference on Computer Vision and Pattern Recognition, Salt Lake City, UT, USA. (2018). p. 4510–20. doi: 10.1109/CVPR.2018.00474

[20] Chollet F. “Xception: Deep learning with depthwise separable convolutions,” In: 2017 IEEE Conference on Computer Vision and Pattern Recognition (CVPR), Honolulu, HI, USA (2017), pp. 1800-1807. doi: 10.1109/CVPR.2017.195

[21] Ge Z Liu S Wang F Li Z Sun J. YOLOX: Exceeding YOLO Series in 2021. arXiv; 2021 (2021). doi: 10.48550/ARXIV.2107.08430

[22] Hirsch FR McElhinny A Stanforth D Ranger-Moore J Jansson M Kulangara K. PD-L1 immunohistochemistry assays for lung cancer: results from phase 1 of the blueprint PD-L1 IHC assay comparison project. J Thorac Oncol. (2017) 12:208–22. doi: 10.1016/j.jtho.2016.11.2228, PMID: 27913228

[23] Crosta S Boldorini R Bono F Brambilla V Dainese E Fusco N. PD-L1 testing and squamous cell carcinoma of the head and neck: A multicenter study on the diagnostic reproducibility of different protocols. Cancers (Basel). (2021) 13:292. doi: 10.3390/cancers13020292, PMID: 33466794 PMC7830149

[24] Bera K Schalper KA Rimm DL Velcheti V Madabhushi A. Artificial intelligence in digital pathology - new tools for diagnosis and precision oncology. Nat Rev Clin Oncol. (2019) 16:703–15. doi: 10.1038/s41571-019-0252-y, PMID: 31399699 PMC6880861

[25] Vandenberghe ME Scott ML Scorer PW Söderberg M Balcerzak D Barker C. Relevance of deep learning to facilitate the diagnosis of HER2 status in breast cancer. Sci Rep. (2017) 7:45938. doi: 10.1038/srep45938, PMID: 28378829 PMC5380996

[26] Wu S Yue M Zhang J Li X Li Z Zhang H. The role of artificial intelligence in accurate interpretation of HER2 immunohistochemical scores 0 and 1+ in breast cancer. Mod Pathol. (2023) 36:100054. doi: 10.1016/j.modpat.2022.100054, PMID: 36788100

[27] Saha M Chakraborty C Arun I Ahmed R Chatterjee S. An advanced deep learning approach for ki-67 stained hotspot detection and proliferation rate scoring for prognostic evaluation of breast cancer. Sci Rep. (2017) 7:3213. doi: 10.1038/s41598-017-03405-5, PMID: 28607456 PMC5468356

[28] Liu J Zheng Q Mu X Zuo Y Xu B Jin Y. Automated tumor proportion score analysis for PD-L1 (22C3) expression in lung squamous cell carcinoma. Sci Rep. (2021) 11:15907. doi: 10.1038/s41598-021-95372-1, PMID: 34354151 PMC8342621

[29] Liu Y Zhen T Fu Y Wang Y He Y Han A. AI-powered segmentation of invasive carcinoma regions in breast cancer immunohistochemical whole-slide images. Cancers (Basel). (2023) 16:167. doi: 10.3390/cancers16010167, PMID: 38201594 PMC10778369

[30] Feng M Deng Y Yang L Jing Q Zhang Z Xu L. Automated quantitative analysis of Ki-67 staining and HE images recognition and registration based on whole tissue sections in breast carcinoma. Diagn Pathol. (2020) 15:65. doi: 10.1186/s13000-020-00957-5, PMID: 32471471 PMC7257511

