# Supplementary material for: Automated interpretation of PD-L1 CPS based on multi-AI models integration strategy in gastric cancer
Source: Front Immunol. 2025 Aug 6;16:1614099. doi: 10.3389/fimmu.2025.1614099 (PMC12364678; doi:10.3389/fimmu.2025.1614099)
Supplement: Supplementary file 1 [file DataSheet1.docx]

**Supplemental Material**


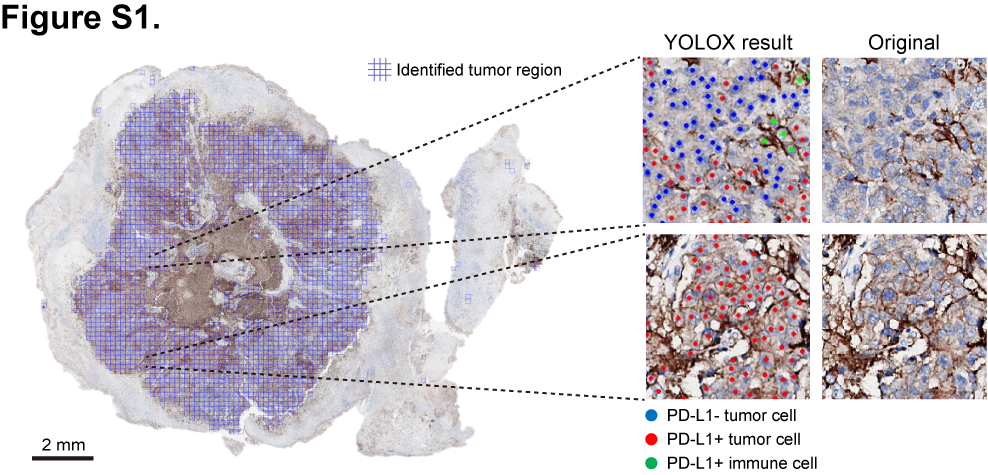


**Figure S1. Schematic representation of cellular identification results using the YOLOX model.**

An example diagram of the cellular identification model results and the corresponding original patch illustrating cell nucleus (DAPI-stained, blue) and PD-L1 expression (brown). Distinct spots denote algorithm-detected cells: red, PD-L1^+^ tumor cells; green, PD-L1^+^ immune cells; and blue, PD-L1^-^ tumor cells.

**
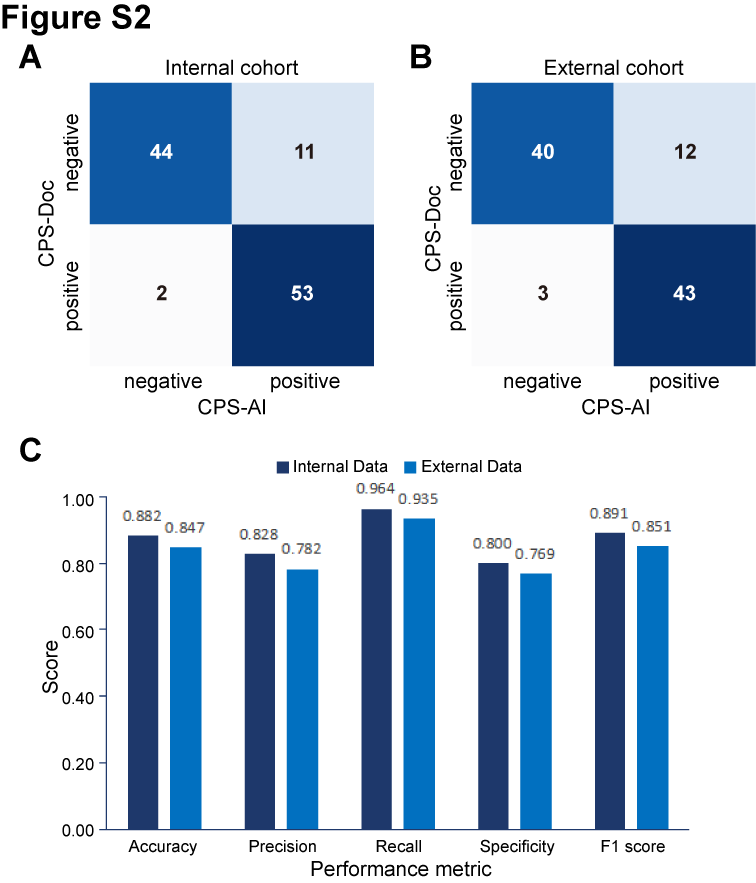
**

**Figure S2. Performance evaluation of the AI pipeline without classification model for combined positve score (CPS) prediction.**

Comparison of CPS prediction results between the AI pipeline without classification model (CPS-AI) and CPS evaluated by pathologists (CPS-Doc) in **(A)** the internal cohort and **(B)** the external cohort. **(C)** Histograms of performances of AI models in internal and external cohorts.

**
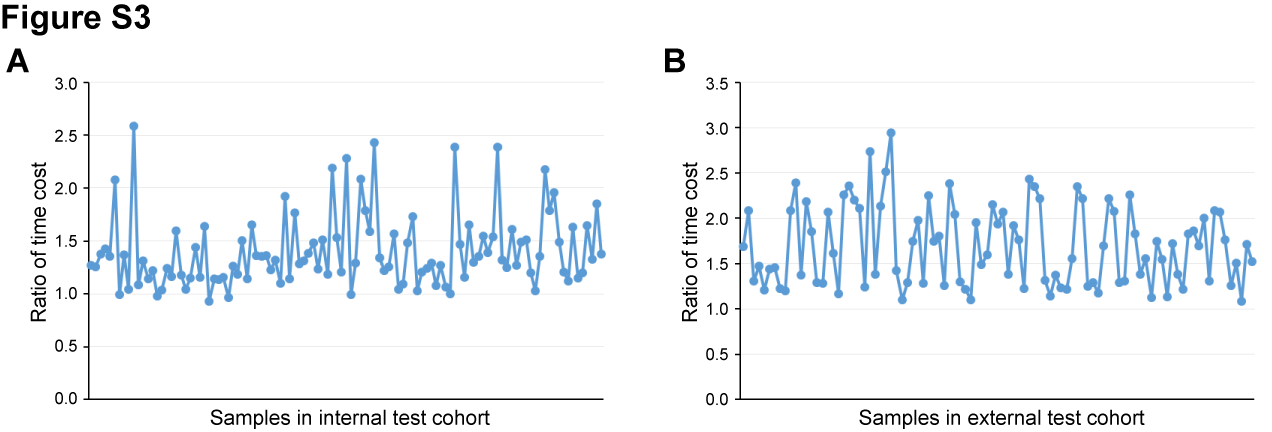
**

**Figure S3. Comparison of time consumption across each sample.**

Each dot represent the ratio of time taken for AI pipeline with the classification model to that without the classification model for each sample in **(A)** the internal cohort and **(B)** the external cohort.
